# Supplementary material for: Modeling glioblastoma heterogeneity as a dynamic network of cell states
Source: Mol Syst Biol. 2021 Sep 16;17(9):e10105. doi: 10.15252/msb.202010105 (PMC8444284; doi:10.15252/msb.202010105)
Supplement: Supplementary file 6 — Source Data for Figure 5 [file MSB-17-e10105-s004.zip › Figure5A_sourcedata/GSEA_3017/nosigdb_stateA.GseaPreranked.1622795026485/RICHARDS_NOWAKOWSKI_SCIENCE_2017_ASTROCYTE_UPREG.html]

Details for gene set RICHARDS\_NOWAKOWSKI\_SCIENCE\_2017\_ASTROCYTE\_UPREG[GSEA]

|  || Dataset | state53017 |
| Phenotype | NoPhenotypeAvailable |
| Upregulated in class | na\_neg |
| GeneSet | RICHARDS\_NOWAKOWSKI\_SCIENCE\_2017\_ASTROCYTE\_UPREG |
| Enrichment Score (ES) | -0.38381425 |
| Normalized Enrichment Score (NES) | -2.5514348 |
| Nominal p-value | 0.0 |
| FDR q-value | 1.1269842E-4 |
| FWER p-Value | 0.002 |
Table: GSEA Results Summary

  

Fig 1: Enrichment plot: RICHARDS\_NOWAKOWSKI\_SCIENCE\_2017\_ASTROCYTE\_UPREG      
 Profile of the Running ES Score & Positions of GeneSet Members on the Rank Ordered List

  

| PROBE | GENE SYMBOL | GENE\_TITLE | RANK IN GENE LIST | RANK METRIC SCORE | RUNNING ES | CORE ENRICHMENT || 1 | FABP5 |  |  | 0 | 1.759 | 0.0260 | No |
| 2 | CREM |  |  | 2 | 1.023 | 0.0400 | No |
| 3 | PDGFRA |  |  | 5 | 0.871 | 0.0505 | No |
| 4 | PCDH10 |  |  | 14 | 0.734 | 0.0520 | No |
| 5 | DBI |  |  | 17 | 0.683 | 0.0598 | No |
| 6 | OLIG2 |  |  | 18 | 0.674 | 0.0697 | No |
| 7 | OLIG1 |  |  | 22 | 0.652 | 0.0759 | No |
| 8 | VIM |  |  | 46 | 0.543 | 0.0569 | No |
| 9 | MMP16 |  |  | 74 | 0.477 | 0.0323 | No |
| 10 | FGFBP3 |  |  | 75 | 0.477 | 0.0394 | No |
| 11 | PRTFDC1 |  |  | 104 | 0.448 | 0.0132 | No |
| 12 | TGIF1 |  |  | 107 | 0.447 | 0.0175 | No |
| 13 | ZEB1 |  |  | 109 | 0.446 | 0.0229 | No |
| 14 | KANK1 |  |  | 132 | 0.419 | 0.0033 | No |
| 15 | S100A16 |  |  | 156 | 0.404 | -0.0177 | No |
| 16 | TNC |  |  | 159 | 0.402 | -0.0141 | No |
| 17 | MAP3K1 |  |  | 167 | 0.396 | -0.0165 | No |
| 18 | PARD3 |  |  | 200 | 0.375 | -0.0484 | No |
| 19 | SERPINE2 |  |  | 201 | 0.373 | -0.0429 | No |
| 20 | EPB41L2 |  |  | 208 | 0.371 | -0.0445 | No |
| 21 | LRRN1 |  |  | 210 | 0.370 | -0.0401 | No |
| 22 | RAB13 |  |  | 220 | 0.366 | -0.0453 | No |
| 23 | SCN1A |  |  | 237 | 0.355 | -0.0588 | No |
| 24 | ALCAM |  |  | 252 | 0.348 | -0.0701 | No |
| 25 | SLC25A5 |  |  | 266 | 0.340 | -0.0803 | No |
| 26 | BCAN |  |  | 276 | 0.334 | -0.0859 | No |
| 27 | MT1X |  |  | 285 | 0.331 | -0.0904 | No |
| 28 | SWAP70 |  |  | 323 | 0.317 | -0.1290 | No |
| 29 | IDI1 |  |  | 334 | 0.314 | -0.1361 | No |
| 30 | ENKUR |  |  | 389 | 0.298 | -0.1950 | No |
| 31 | ATP13A4 |  |  | 410 | 0.290 | -0.2142 | No |
| 32 | DOCK1 |  |  | 414 | 0.289 | -0.2134 | No |
| 33 | ABLIM1 |  |  | 452 | 0.280 | -0.2526 | No |
| 34 | BCHE |  |  | 456 | 0.280 | -0.2520 | No |
| 35 | SLITRK2 |  |  | 458 | 0.279 | -0.2490 | No |
| 36 | ABI1 |  |  | 459 | 0.279 | -0.2449 | No |
| 37 | MT2A |  |  | 473 | 0.275 | -0.2561 | No |
| 38 | PSAT1 |  |  | 491 | 0.271 | -0.2720 | No |
| 39 | ZFP36L1 |  |  | 532 | 0.264 | -0.3150 | No |
| 40 | ITGA6 |  |  | 544 | 0.261 | -0.3240 | No |
| 41 | VEGFA |  |  | 546 | 0.261 | -0.3213 | No |
| 42 | DSEL |  |  | 559 | 0.258 | -0.3316 | No |
| 43 | SOX9 |  |  | 577 | 0.254 | -0.3478 | No |
| 44 | AGT |  |  | 587 | 0.252 | -0.3546 | No |
| 45 | ITGAV |  |  | 599 | -0.250 | -0.3638 | No |
| 46 | CD99 |  |  | 604 | -0.252 | -0.3647 | No |
| 47 | SYPL1 |  |  | 608 | -0.255 | -0.3645 | No |
| 48 | NLGN4X |  |  | 613 | -0.259 | -0.3653 | No |
| 49 | ABAT |  |  | 617 | -0.259 | -0.3650 | No |
| 50 | DAG1 |  |  | 622 | -0.260 | -0.3659 | No |
| 51 | TACC1 |  |  | 623 | -0.260 | -0.3620 | No |
| 52 | GPR37L1 |  |  | 624 | -0.261 | -0.3582 | No |
| 53 | ARHGEF26 |  |  | 628 | -0.262 | -0.3578 | No |
| 54 | KCNJ16 |  |  | 634 | -0.264 | -0.3598 | No |
| 55 | CAMK2D |  |  | 635 | -0.264 | -0.3559 | No |
| 56 | SDC3 |  |  | 637 | -0.264 | -0.3531 | No |
| 57 | GNG12 |  |  | 640 | -0.265 | -0.3515 | No |
| 58 | MAGI1 |  |  | 642 | -0.266 | -0.3488 | No |
| 59 | PTPRA |  |  | 643 | -0.267 | -0.3448 | No |
| 60 | ARHGEF6 |  |  | 659 | -0.273 | -0.3584 | No |
| 61 | NOTCH1 |  |  | 666 | -0.280 | -0.3613 | No |
| 62 | PEA15 |  |  | 668 | -0.281 | -0.3583 | No |
| 63 | SPRY2 |  |  | 669 | -0.282 | -0.3541 | No |
| 64 | SEMA6A |  |  | 679 | -0.292 | -0.3604 | No |
| 65 | RHOJ |  |  | 682 | -0.293 | -0.3584 | No |
| 66 | DDR1 |  |  | 683 | -0.294 | -0.3540 | No |
| 67 | S100B |  |  | 696 | -0.299 | -0.3637 | No |
| 68 | MALAT1 |  |  | 702 | -0.302 | -0.3651 | No |
| 69 | ARAP2 |  |  | 719 | -0.315 | -0.3792 | Yes |
| 70 | PCDH9 |  |  | 720 | -0.315 | -0.3745 | Yes |
| 71 | ID2 |  |  | 727 | -0.323 | -0.3768 | Yes |
| 72 | SCD5 |  |  | 729 | -0.324 | -0.3731 | Yes |
| 73 | C1orf61 |  |  | 733 | -0.328 | -0.3718 | Yes |
| 74 | ELOVL2 |  |  | 735 | -0.329 | -0.3681 | Yes |
| 75 | HES1 |  |  | 736 | -0.331 | -0.3632 | Yes |
| 76 | TIMP2 |  |  | 737 | -0.332 | -0.3583 | Yes |
| 77 | PON2 |  |  | 743 | -0.338 | -0.3592 | Yes |
| 78 | JAG1 |  |  | 745 | -0.339 | -0.3553 | Yes |
| 79 | MYO6 |  |  | 750 | -0.343 | -0.3549 | Yes |
| 80 | HEY1 |  |  | 751 | -0.344 | -0.3498 | Yes |
| 81 | WSCD1 |  |  | 761 | -0.351 | -0.3552 | Yes |
| 82 | NTM |  |  | 762 | -0.354 | -0.3500 | Yes |
| 83 | SLC4A4 |  |  | 763 | -0.355 | -0.3447 | Yes |
| 84 | MPC1 |  |  | 769 | -0.360 | -0.3453 | Yes |
| 85 | ITGB8 |  |  | 774 | -0.363 | -0.3446 | Yes |
| 86 | MLC1 |  |  | 783 | -0.369 | -0.3485 | Yes |
| 87 | MSMO1 |  |  | 803 | -0.386 | -0.3651 | Yes |
| 88 | CXXC5 |  |  | 809 | -0.391 | -0.3651 | Yes |
| 89 | FHL1 |  |  | 814 | -0.397 | -0.3639 | Yes |
| 90 | LTBP1 |  |  | 815 | -0.397 | -0.3581 | Yes |
| 91 | DST |  |  | 818 | -0.400 | -0.3545 | Yes |
| 92 | HOPX |  |  | 821 | -0.403 | -0.3509 | Yes |
| 93 | IGFBP2 |  |  | 822 | -0.404 | -0.3449 | Yes |
| 94 | GJA1 |  |  | 823 | -0.405 | -0.3389 | Yes |
| 95 | DDAH1 |  |  | 828 | -0.413 | -0.3375 | Yes |
| 96 | PLOD2 |  |  | 838 | -0.431 | -0.3417 | Yes |
| 97 | NACC2 |  |  | 839 | -0.431 | -0.3353 | Yes |
| 98 | TGFB2 |  |  | 840 | -0.432 | -0.3289 | Yes |
| 99 | CHPT1 |  |  | 844 | -0.437 | -0.3259 | Yes |
| 100 | ZFP36L2 |  |  | 848 | -0.443 | -0.3229 | Yes |
| 101 | APC |  |  | 856 | -0.447 | -0.3245 | Yes |
| 102 | DCLK2 |  |  | 859 | -0.449 | -0.3202 | Yes |
| 103 | SLC1A2 |  |  | 860 | -0.450 | -0.3135 | Yes |
| 104 | SLC35F1 |  |  | 871 | -0.482 | -0.3181 | Yes |
| 105 | JUN |  |  | 886 | -0.525 | -0.3268 | Yes |
| 106 | F3 |  |  | 888 | -0.526 | -0.3202 | Yes |
| 107 | UBL3 |  |  | 893 | -0.535 | -0.3170 | Yes |
| 108 | GPC6 |  |  | 896 | -0.540 | -0.3113 | Yes |
| 109 | GPM6B |  |  | 897 | -0.542 | -0.3033 | Yes |
| 110 | ATP1B2 |  |  | 904 | -0.558 | -0.3021 | Yes |
| 111 | MT3 |  |  | 905 | -0.559 | -0.2938 | Yes |
| 112 | SAT1 |  |  | 909 | -0.573 | -0.2888 | Yes |
| 113 | RAB31 |  |  | 916 | -0.586 | -0.2872 | Yes |
| 114 | GLUL |  |  | 920 | -0.612 | -0.2817 | Yes |
| 115 | ZMAT3 |  |  | 929 | -0.650 | -0.2814 | Yes |
| 116 | ID1 |  |  | 931 | -0.651 | -0.2730 | Yes |
| 117 | TTYH1 |  |  | 932 | -0.652 | -0.2633 | Yes |
| 118 | CST3 |  |  | 933 | -0.680 | -0.2533 | Yes |
| 119 | CLU |  |  | 938 | -0.685 | -0.2478 | Yes |
| 120 | GRIA4 |  |  | 941 | -0.698 | -0.2398 | Yes |
| 121 | HEPACAM |  |  | 943 | -0.709 | -0.2305 | Yes |
| 122 | RNF180 |  |  | 945 | -0.725 | -0.2210 | Yes |
| 123 | LRRC17 |  |  | 949 | -0.769 | -0.2131 | Yes |
| 124 | LYPD1 |  |  | 955 | -0.839 | -0.2066 | Yes |
| 125 | CNN3 |  |  | 961 | -0.883 | -0.1993 | Yes |
| 126 | ZBTB20 |  |  | 964 | -0.895 | -0.1884 | Yes |
| 127 | FABP7 |  |  | 971 | -0.994 | -0.1808 | Yes |
| 128 | SLC1A3 |  |  | 973 | -1.097 | -0.1657 | Yes |
| 129 | LRRN3 |  |  | 977 | -1.233 | -0.1510 | Yes |
| 130 | CYR61 |  |  | 978 | -1.296 | -0.1318 | Yes |
| 131 | PMP22 |  |  | 982 | -1.519 | -0.1129 | Yes |
| 132 | IGFBP5 |  |  | 983 | -1.595 | -0.0893 | Yes |
| 133 | SPARCL1 |  |  | 984 | -1.810 | -0.0625 | Yes |
| 134 | NEAT1 |  |  | 986 | -2.049 | -0.0334 | Yes |
| 135 | GFAP |  |  | 987 | -2.255 | 0.0000 | Yes |
Table: GSEA details [plain text format]

  

Fig 2: RICHARDS\_NOWAKOWSKI\_SCIENCE\_2017\_ASTROCYTE\_UPREG: Random ES distribution      
 Gene set null distribution of ES for **RICHARDS\_NOWAKOWSKI\_SCIENCE\_2017\_ASTROCYTE\_UPREG**
